# Uniparental Markers of Contemporary Italian Population Reveals Details on Its Pre-Roman Heritage

**DOI:** 10.1371/journal.pone.0050794

**Published:** 2012-12-10

**Authors:** Francesca Brisighelli, Vanesa Álvarez-Iglesias, Manuel Fondevila, Alejandro Blanco-Verea, Ángel Carracedo, Vincenzo L. Pascali, Cristian Capelli, Antonio Salas

**Affiliations:** 1 Unidade de Xenética, Facultade de Medicina, Instituto de Medicina Legal, Universidade de Santiago de Compostela, Galicia, Spain; 2 Forensic Genetics Laboratory, Institute of Legal Medicine, Università Cattolica del Sacro Cuore, Rome, Italy; 3 Department of Zoology, University of Oxford, Oxford, United Kingdom; 4 Fundación Pública Galega de Medicina Xenómica (FPGMX-SERGAS), CIBER enfermedades raras, Santiago de Compostela, Galicia, Spain; University of Florence, Italy

## Abstract

**Background:**

According to archaeological records and historical documentation, Italy has been a melting point for populations of different geographical and ethnic matrices. Although Italy has been a favorite subject for numerous population genetic studies, genetic patterns have never been analyzed comprehensively, including uniparental and autosomal markers throughout the country.

**Methods/Principal Findings:**

A total of 583 individuals were sampled from across the Italian Peninsula, from ten distant (if homogeneous by language) ethnic communities — and from two linguistic isolates (Ladins, Grecani Salentini). All samples were first typed for the mitochondrial DNA (mtDNA) control region and selected coding region SNPs (mtSNPs). This data was pooled for analysis with 3,778 mtDNA control-region profiles collected from the literature. Secondly, a set of Y-chromosome SNPs and STRs were also analyzed in 479 individuals together with a panel of autosomal ancestry informative markers (AIMs) from 441 samples. The resulting genetic record reveals clines of genetic frequencies laid according to the latitude slant along continental Italy – probably generated by demographical events dating back to the Neolithic. The Ladins showed distinctive, if more recent structure. The Neolithic contribution was estimated for the Y-chromosome as 14.5% and for mtDNA as 10.5%. Y-chromosome data showed larger differentiation between North, Center and South than mtDNA. AIMs detected a minor sub-Saharan component; this is however higher than for other European non-Mediterranean populations. The same signal of sub-Saharan heritage was also evident in uniparental markers.

**Conclusions/Significance:**

Italy shows patterns of molecular variation mirroring other European countries, although some heterogeneity exists based on different analysis and molecular markers. From North to South, Italy shows clinal patterns that were most likely modulated during Neolithic times.

## Introduction

Italy has historically been a convenient destination for human populations migrating from Africa, the Middle East and European locations, in part due to the geomorphological characteristics of the Italian Peninsula [1]. These groups settled preferentially on the islands and coastal territories [1] 500,000 years ago (ya), that is, along the Lower Paleolithic, the longest period of human prehistory, which was dominated by the notable diffusion of tools made from flaked stone [2]. Although rich in tools and animal bones, only some of these sites have provided a small quantity of human skeletal remains resembling those from the more recent sites of the Middle Paleolithic, dating to the Riss-Würm interglacial period and part of the succeeding Würm glaciation (*circa* 120,000 to 36,000 ya). These bones belong to a species named *Homo sapiens neanderthalensis*. [2] In this long Paleolithic period, navigation across the Mediterranean was probably rare and some present-day islands were accessible across land bridges later covered by the rising sea [3]. During the Upper Paleolithic, from 36,000 to 10,000 ya, the icecap expansion of the Late Glacial Maximum (LGM) pushed southward groups of hunters living in Central European areas [1], and the Neanderthals gave way to the present species of man *Homo sapiens sapiens* during the final phases of the Würm glaciation. The numerous traces from this period are particularly rich in burials, animal bones and tools, the latter having been worked with increased precision [2]. In the few thousand years of the following Mesolithic period (*circa* 10,000 to 6,000 ya) the climate continued to grow milder and sites from this period have been found throughout the entire Italian peninsula, being along the coasts in the plains and on the mountains. With the Neolithic period, from *circa* 6,000 BC to 2,800 BC, the traditional hunting and gathering economy was replaced by the introduction of agriculture, stock rearing, weaving and pottery. This new cultural influence came particularly from the Eastern Mediterranean and the Near East. Using the pottery production above all, it has been possible to reconstruct fairly accurately the various phases of this complex period [2]. During the Copper, Bronze and Iron ages, numerous population movements occurred between the Mediterranean basin and the Middle East [4]. Exchange of metals would determine the transformation of the first social organizations in ancient civilizations [4]. Sardinia, Sicily and Tuscany were among the first Italian territories to be occupied by humans due to their strategic location and the presence in their territories of important metal resources [5].

Different cultures, recognized on the basis of different archeological findings, settlements and burial traditions, arose in the period between the Mesolithic and Iron Age. Before the Roman conquest, ancient Italy was characterized only by the presence of Indo-European populations [6] living in the Italian Peninsula since the second millennium BC, corresponding to the period between the Iron Age and Romanization [2]. During all this period there were also increasing contacts with the Phoenician and Greek colonists: the former being largely present on the coasts of Sardinia and western Sicily and the latter in Southern Italy. These colonies had a considerable influence on the development of local cultures (from the Picenian to Campano-Samnite and the Apulian to Bruttio-Lucanian) [5].

The record of all the populations that inhabited the Italian territory during (pre)-history is incomplete; many records were of uncertain location and/or ambiguous denomination [6]. At the beginning of the first millennium BC the following native tribes could be distinguished on the Italian territory: the Ligures, on the coast that bears their name, in the northern Apennine valleys, part of the pre-alpine valleys and the western Po Valley; the Sicani, in the interior of Sicily; and the Itali, in present-day Calabria (from whom comes the name ‘Italy’, which was to be extended to all the territory of the peninsula). Besides the already mentioned Terramare tribe, on the southern edge of the Po Valley, and the Villanovans, probably from Eastern Europe who settled throughout Central Italy, there were also the Umbrians to the east of the upper basin of the Tiber. The Veneti, who occupied the territory that still bears their name, originally came from Illyria as did the Messapii (now modern Salento or South Apulia) and Iapyges, who settled in present-day Puglia (Apulia) [5]. Many other populations of Central-Southern Italy were created by the mixing of local and foreign elements dating back to the previous millennium; it is the case of the Sabines and Latini who settled in Lazio together with Falisci, Aequi, Volsci, Hernici and Ausones. The interior of Abruzzo was dominated by the Vestini, Paeligni and Marsi, while the central Adriatic coast was populated by Picentes, Marrucini and Frentani. The Apennine area of Molise and Basilicata was peopled by the Samnites and Lucanians. In Calabria and Sicily there were also the Bruttii and Siculi.

The Phoenician colonization of the coasts of the Western Mediterranean were mainly limited in Italy to Sardinia and western Sicily and preceded that of the Greeks. It was followed by Punic settlements (Trapani, Palermo, Cagliari) linked to the ancient Phoenician colony of Carthage.

At the time of the Roman Empire, at least two non-Indo-European populations still inhabited Italy, namely, the Ligures, in the northwestern area, and the Etruscans with settlements located in areas far from the Etruria (Tuscany and High Latium), such as the Po Plain and the coast of *Campania*. At the same time, Sardinia experienced the flourishing of a non-Indo-European Nuragic civilization and, then, the Phoenician colonization.

Genetics alone cannot disentangle the extremely complex demography of Italy through history. Some demographic movements have however left signals on uniparental and nuclear markers. Most of the genetic studies targeted local, e.g. [7], or regional, e.g. [8]–[11], Italian populations.

For the Y-chromosome, some attempts have been undertaken to analyze Italian variation to a more general scale [12]–[14]. Many studies have analyzed specific haplogroups in the Y-chromosomes, e.g. [15], [16], or the mtDNA, e.g. [8], [9]. In general, the different studies indicate that the genetic structure of the present Italian population seems to reflect, at least in part, the ethnic stratification of pre-Roman times [14]. Studies carried out in the past appear to show a major North–South cline consistent with archaeological estimates of two distinct processes: the first colonization of the area during the Paleolithic period and the subsequent Neolithic expansion from the Middle East after the last glacial [14]. There is some correspondence between patterns of variation at the Y-chromosome and geography. Thus, northern Italy shows similar frequencies as the haplogroups of Central Europe, with prevalence of the western R1-M173 haplogroup compare to the eastern I-M170. In the North, E3b1-M35 and J2-M172 show low frequencies but are more prevalent in the South, which has been interpreted to be a signal of the gene flow coming from Central European Neolithic farmers [17]. R1a1-M17 is rather rare, both in the North, where it probably originates from eastern Europe, and in the South, of possible Greek provenience [17]. Occurrence of J2-M172 Y-chromosomes in Tuscany has been related to the Etruscan heritage of the region (see [17]). The two Italian major islands, Sicily and Sardinia, show a different demographic history. The Y-chromosome variability of Sicily shares a common history with that of southern Italy, enriched by an additional Arab contribution, but also North African and Greek influences [18]. On the other hand, Sardinia has been considered to be a genetic outlier within Europe showing clear signals of founder effects; some scholars suggest that its peoples could be of ancient Iberian origin [19]; recent genetic studies point to genetic contribution coming from southern France [20].

On the other hand, mitochondrial DNA studies show that Italy does not differ too much from other European populations; however, some populations have the same peculiarities and preserve signals of the ancient past demographic event, such as the Tuscans [8], [9], or the Ladins [7], [21], [22]. Recently, patterns of variation observed in haplogroup U5b3 demonstrated for the first time the existence of a North Italian pre-historical human refuge from the hostile Central European regions covered by the ice of the Last Glacial Maximum period [20]; this area, as was also the Franco-Cantabrian region [23]–[26], served as a region of European repopulation during the beginning of the Holocene.

The main aim of the present study was comprehensively to analyze the patterns of mtDNA and Y-chromosome variation in Italy. This study differs from previous ones in that: (1) it provides mtDNA data from 12 new sample populations from Italy; (2) we analyzed two linguistic isolates, Ladin and Grecani Salentini, the latter sampled for the first time in this study; (3) we analyzed a sample population from Lucera (Southern Italy) for the first time, a population that according to documentation received an important input of North African immigrants during the thirteenth century; (4) we analyzed the patterns of mtDNA variation in Italy globally, that is, by combining more than 3,700 control region profiles from the literature (41 population samples in total) coupled with the more than 580 new profiles provided here; (5) Y-chromosome haplotype and haplogroup patterns are analyzed in parallel with the mtDNA data in order to determine the possible differences that occurred historically in the male *versus* female demographic movements; and (6) the influx of migrants from Africa (North and sub-Saharan) and other regions is also analyzed using phylogeographic inferences, and also a model of admixture based on haplotypic data and a panel of ancestry informative markers (AIMs).

## Materials and Methods

### Ethics statement

Written informed consent was obtained from all sample donors. Analysis of mtDNA sequences was approved by the institutional review boards of the Università Cattolica del Sacro Cuore (Roma). Moreover, the study conforms to the Spanish Law for Biomedical Research (Law 14/2007- 3 of July).

### Samples

**Figure 1:**
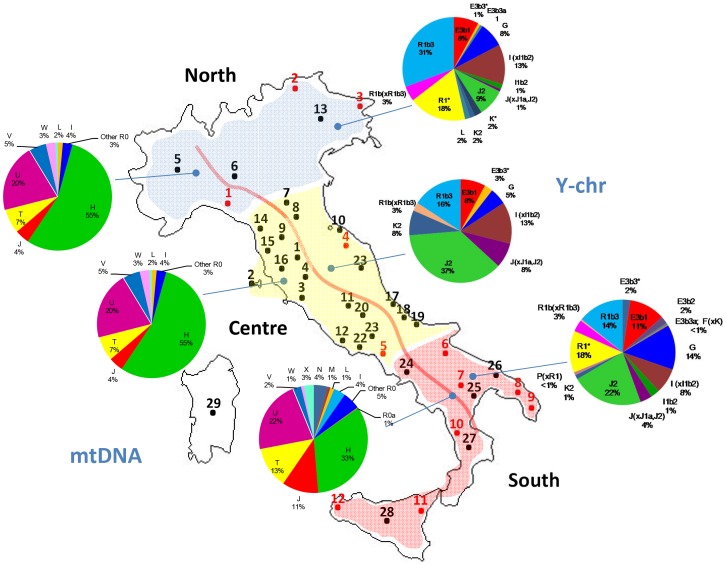
Map showing the location of the samples analyzed in the present study and those collected from the literature (see **Table 1**). Pie charts on the left display the distribution of mtDNA haplogroup frequencies, and those on the right the Y-chromosome haplogroup frequencies.

A total of 583 individuals were sampled from along the Italian Peninsula, representing 12 different populations (**Figure 1**), two of them (Ladin and Grecani Salentini) being linguistic isolates, and the Lucera being a historical enclave of Arabs coming from North Africa. A brief description of these latter three populations is given below.

In the Italian territory, the Alpine arc represents one of the main areas of presence of alloglot populations, some of them biologically isolated for historical and geographic reasons [27]. At the end of the medieval period (∼1200 AD) and especially in the valley zone, a first colonization of native peasants began, starting with the use of lands previously exploited only for pasture and the lumber. Successively, with different modalities and under the control of laic and ecclesiastical owners, the colonization process involved migrant nuclei from the Tyrol, Carinthian area and other zones [28]. Currently, the Alpine arc populations are differentiated with a remarkable cultural diversity that is well represented by linguistic elements. Thus, besides the official main languages, numerous minority languages or dialects are also the cultural patrimony of linguistic minorities [27], [29]. Ladin is often attributed to be a relic of vulgar Latin dialects associated with Rhaeto-Romance languages. In the vast multi-ethnic Holy Roman Empire, and then after 1804 the Austrian empire, the Ladins were left in relative peace and were allowed to continue the use of their language and culture.

Grecani Salentini is a Hellenic-speaking linguistic island of Salento, situated in southern Puglia, and consisting of nine municipalities in which a neo-Greek dialect, also known as Grecanic or Griko, is spoken. The origins of this linguistic island in Salentine Greece are uncertain. The German linguist G. Rohlfs proposed its origin in the *Magna Graecia* region; while O. Parlangeli suggests a Byzantine derivation of the Griki of Salento. Greek researchers (e.g. A. Karanastasis) claim the input of Byzantine elements in the pre-existing *Magna Graecia* matrix. The Greek arrival in the Salentine Peninsula occurred both in the *Magna Graecia*, and posterior Byzantine dominations. The numerous villages of Grecani Salentini had a Greek culture and language and practiced the Greek-orthodox religion. In the beginning of the Norman conquest (eleventh century), and more intensively with the arrival of different *casati* (clans) (Svevian, Angioin, Aragones, etc), the catholic clergy supplanted those of the orthodox faith [30].

The Lucera population has received an important influx from North African Arab peoples (see [31]). Thus, after the collapse of the Roman Empire in Europe, the Arab domination spread into the Mediterranean Basin. Referred to either as Moors in Iberia or Saracens in Southern Italy and Sicily, Arabs arrived in Europe in 711 AD, and in 831 AD Iberia and Sicily were almost completely subjected to Arab domination [31]. In the thirteenth century, Frederick II moved the Sicilian Arabs to the city of Lucera (North Apulia) [32]. This sample was genotyped for STRs and Y-chromosome SNPs in Capelli et al. [31]

To the best of our knowledge, all individuals collected in the present study were not maternally and paternally closely related; they had different surnames and all the donors referred back at least two generations in the region where the samples were collected.

All the samples were analyzed for the control region and selected mtSNPs (see below). A subset of the samples comprised unrelated males (*n* = 292) representing seven different populations. These samples were genotyped for a panel of 17 Y-chromosome SNPs (see below), and were previously genotyped for the Yfiler [33]. In addition, autosomal ancestry informative markers (AIMs) were genotyped in 441 individuals (see below).

### DNA extraction

Blood extraction was performed with a salting-out method [34], modified and re-adapted to buccal cells. Swabs were incubated in 500 µl of 0.2 sodium acetate, 35 µl of 10% SDS and 20 µl of 20 mg/ml Proteinase K for 16 hours at 56°C. They were then removed and 500 µl of 3 M NaCl solution was added. Proteins were removed by centrifugation, and the DNA precipitated by adding 1 ml of ethanol 100% at −20°C for a few hours. After centrifugation, the DNA pellet was twice washed with ethanol 70%, dried and re-suspended in water. For the blood samples, aliquots of 500 µl each were thawed and red cells selectively lysed by a 1× lysis buffer. After three washes with the lysis buffer, white cells were pelleted and the DNA extracted using the salting-out protocol. All the samples were quantified by direct comparison with standard on agarose 1% minigels (1 g of agarose in 100 ml of TBE 1X- from the 1∶10 diluition of TBE 10X).

### PCR and mtDNA control region sequencing

MtDNA has been sequenced for the complete control region, from position 16024 (in HVS-I) to 569 (in HVS-II). The first and second hypervariable regions (HVS-I/II) were amplified *via* the polymerase chain reaction (PCR) and using primers reported by Álvarez-Iglesias et al. [35].

PCR was carried out in a 25 µl reaction mix with 1× reaction buffer (20 mM Tris-HCl, ph 8.0, 0.1 mM EDTA, 1 mM DDT, 50% (v/v) glycerol), 1.5 mM MgCl_2_, 200 mM each dNTP, 0.4 µM each primer, 2.5 U (Units). Taq polymerase and 0.1–1 ng DNA template was added to the reaction mixture (*Taq* DNA Polymerase, recombinant. INVITROGEN® Corporation). Amplification was carried out in a GENE AMP® PCR SYSTEM 9700 (Applied Biosystems, Foster City, California,U.S.A.) using a hot start at 95°C for 1 min, followed by 36 cycles at 95°C for 30 sec, 55°C for 60 sec, and 72°C for 30 sec and a final extension at 72°C for 15 min. Before the sequencing reaction, PCR products were checked by electrophoresis in polyacrylamide non-denaturing gel (T9, C5), and subsequently the gel was stained with silver nitrate. PCR products were then purified with a MultiScreen® PCR_μ96_ Plate (Millipore, Bedford, Ma 01730, U.S.A), 96-well device.The vacuum-based, size exclusion separation effectively and quickly removed the containing salts, unincorporated dNTPs and primers from PCR reactions. Cycle sequencing was performed on both strands in a GENE AMP® PCR SYSTEM 9700 (AB) thermal cycler using the ABI Prism® dRhodamine Terminator Cycle Sequencing Ready Reaction Kit (AB). This kit consists of a reaction mix composed of: DNA-modified and termostable polymerase, Buffer Tris-HCl (pH 9.0), MgCl_2_, dNTPs, dichlororhodamine-marked ddNTPs. An aliquote of 30 ng amplicon and 3.2 ρM primers were added to a 2 µl reaction mix. Sequencing was carried out using a hot start at 96°C for 4 min, followed by 36 cycles at 96°C for 15 sec, 50°C for 10 sec, 60°C for 2 sec and a final extension at 60°C for 10 min. The removal of excess dideoxy terminators, primers and buffer was accomplished with an alcoholic purification.

The sequence products were denatured with deionized formamide and analyzed by capillary electrophoresis on an ABI PRISM 3130® Genetic Analyzer (AB).The resulting data were analyzed with PE/ABD software Sequencing Analysis 5.2 and sequences were aligned and compared with the Cambridge sequence [36] from position 16024 to16569 for HVS-I and from position 1 to 600 for HVS-II by the SeqScape v.2.0 (AB).

### Analysis of mtDNA coding region SNPs

Biallelic markers were genotyped using a multiplex approach [37]. The selected SNPs were combined into two multiplex reactions. Multiplex 1 included a selection of SNPs defining common European haplogroups [38]. Multiplex 2 included exclusively polymorphisms defining sub-lineages inside haplogroup H. Primers were designed in order to adjust the annealing temperatures and amplicon lengths to allow analysis in multiplex reactions [37]. The sizes of the PCR products ranged from 80 to 224 bp.

Both multiplexes were performed using 10 ng of DNA template in a 25 µl reaction volume comprising 1× Taq Gold Buffer (AB), 200 µM of each dNTP, 2 mM MgCl_2_ and 0.5 U of AmpliTaq Gold Polymerase (AB). For the primer concentrations, see [37].

Amplification was carried out using a GENE AMP® PCR SYSTEM 9700 (AB) thermocycler. After a 95°C pre-incubation step for 11 min, PCR was performed for a total of 32 cycles using the following conditions: 94°C denaturation for 30 sec, annealing at 60°C for 30 sec and extension at 72°C for 1 min, followed by a 15 min final extension at 72°C. PCR products were checked by polyacrylamide gel electrophoresis (T9, C5) visualized by silver staining.

After amplification, PCR products required purification to remove primers and unincorporated dNTPs. Post-PCR purification was performed with ExoSapIT (Amershan Pharmacia Biotech): 1 µl of PCR product was incubated with 0.5 µl of ExoSapIT for 15 min at 37°C followed by 15 min at 80°C for enzyme inactivation. The minisequencing reaction was performed in a GENE AMP® PCR SYSTEM 9700 (AB) thermocycler following the recommendations of the manufacturer: 2 µl of SNaPshot ready reaction mix, 0.2 µM of extension primer for each SNP (see [37]) and 1 µl of both purified PCR products in a total volume of 7 µl. The reaction mixture was subjected to 25 single base extension cycles of denaturation at 96°C for 10 sec, annealing at 50°C for 5 sec and with an extension at 60°C during 30 sec. After minisequencing reactions, a post-extension treatment to remove the 5′-phosphoryl group of ddNTPs aided the prevention of co-migration of unincorporated ddNTPs with extended primers and production of a high background signal. The final volume (7 µl) was treated with 0.7 µl of SAP (Amersham Biosciences) for 60 min at 37°C, followed by 15 min at 80°C for enzyme inactivation.

The minisequencing products (1.5 µl) were mixed with 10 µl of HiDi™ formamide and 0.2 µl of GeneScan-120 LIZ size standard (AB) and electroforesis was performed on an ABI PRISM 3130® Genetic Analyser (AB). The resulting data was analyzed with Gene Mapper ID.

### Minisequencing of SNPs characterizing additional typical European haplogroups

Samples that were determined (using the SNP panel above) as being derived from J/T (T14766C; C7028T; T4216C), U (T14766C; C7028T; A12308G) and the U-subclade K (T14766C; C7028T; A12308G; A10398G), were further genotyped using an additional set of 14 haplogroup-specific SNP markers that identify the following sub-branches: J1 (G3010A), J1b (G3010A; C13879T), J1c (G3010A; C114798T), J2 (G15257A), T2a (A14687G), T2b (G5147A), U5a (A14793G), U5a1 (A14793G; A15218G), U5b (A7768G), U5b1 (A7768G; A5656G), U5b2 (A7768G; C1721T), K1 (T14798C; T1189C), K1a (T14798C; T1189C; C0497T) and K2 (T14798C; T1189C; T9716C). PCR and minisequencing reactions were performed as described above. For PCR and minisequencing primer concentrations, see **Table S1**.

### Genotyping of Y-SNPs

Biallelic markers were genotyped using a multiplex approach [39]. A set of 30 SNPs was tested, allowing assignation of the analyzed Y-chromosome to haplogroups (Hg), following the nomenclature and the phylogenetic relationships defined from the Y Chromosome Consortium [40]. The selected method for allele discrimination was a single base extension reaction using the SNaPshot multiplex kit (AB). We added the M269 marker to the first of the four multiplexes, in order better to dissect the sub-haplogroup R1b (R1b3). The primers of this marker were M269-F 5′-TCA TGC CTA GCC TCA TTC CT-3′ and M269-R 5′-TCT TTT GTG TGC CTT CTG AGG-3′, and the minisequencing primer 5′-GGA ATG ATC AGG GTT TGG TTA AT-3′.

### Genotyping of AIMs

**Figure 2:**
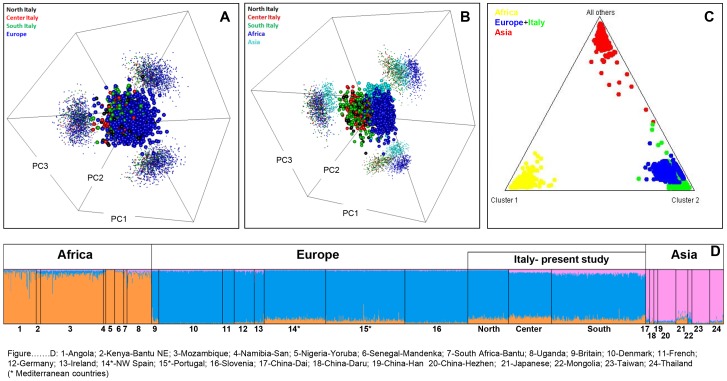
Analysis of AIMs in Italian populations *versus* other continental population groups. (A) PCA of Italian populations divided into the main regions North, Center and South (as analyzed in the present study) and other European populations; (B) the same Italian populations plus sub-Saharan African, and Asian populations; (C) triangle plot as obtained using STRUCTURE analysis of Italian, European, sub-Saharan, and Asian populations; (D) bar plot of ancestral membership values as obtained using STRUCTURE analysis of the same populations used in (C). Population codes: 1: Angola; 2: Kenya-Bantu NE; 3: Mozambique; 4: Namibia-San; 5: Nigeria-Yoruba; 6: Senegal-Mandenka; 7: South Africa-Bantu; 8: Uganda; 9: Britain; 10: Denmark; 11: French; 12: Germany; 13: Ireland; 14*: NW Spain; 15*: Portugal; 16: Slovenia; 17: China-Dai; 18: China-Daru; 19: China-Han; 20: China-Hezhen; 21: Japanese; 22: Mongolia; 23: Taiwan; 24: Thailand. Genotypes were downloaded using the method in [43], [83] and belong to the CEPH panel. An asterisk indicates Mediterranean populations.

A panel of 52 AIMs were genotyped according to Sánchez et al. [41] in a subset of 441 individuals. Several other population datasets were used for inter-population comparisons. This data corresponded to the CEPH panel (http://www.cephb.fr/en/cephdb/) as reported in HapMap (http://hapmap.ncbi.nlm.nih.gov/) and was collected using the data-mining tool SPSmart [42], [43]; it includes population samples from all over the world (Africa, Europe, Asia, etc.); see legend of **Figure 2** for more information.

### Statistical analysis

A total of 42 Italian population samples were analyzed for mtDNA in the present study. Comparative inter-population analyses were also carried out for the HVS-I segment ranging from 16024 to 16365, since this is the analyzed segment common to all of them. Haplotype (*H*) and nucleotide diversity (*π*) and other diversity indices [44]–[46] were computed using DnaSP 4.10.3 software [47]. Problematic variation located around 16189, usually associated to length heteroplasmy e.g. 16182C or 16183C, was ignored. Analysis of molecular variance (AMOVA) was carried out using Arlequin 3.5. [48]. Nomenclature of mtDNA lineages followed previous studies e.g. [23], [25], [38], [49], [50]; see Phylotree for a compilation of the worldwide phylogeny and an update of the nomenclature based on entire mtDNA genomes [51]. Genotyping and documentation errors were monitored following the phylpogenetic principles previously applied e.g. [52]–[59].

Mitochondrial DNA and Y-chromosome data was collected from the literature. The mtDNA data generated in the present study was analyzed together with 3,834 mtDNA HVS-I Italian profiles collected from the literature (**Table S2**; 76 sample populations). The Y-SNPs were analyzed together with 1,251 Italian profiles reported in the literature (16 population samples). A full list of references for all the data used in the present study is given in **Table S2**.

Haplogroup frequencies were estimated by chromosome counting. Statistical differences in haplogroup frequencies were evaluated using a Pearson's chi- square test and by setting up the nominal significant value α as 0.05.

Finally, classification of mtDNA sequences into haplogroups was performed following phylogenetic criteria (Phylotree Build 14, http://www.phylotree.org/) and using both the control region sequence profile and mtSNPs.

## Results

### Molecular diversity of mtDNA and Y-chromosome Italian profiles

Diversity indices were computed for all the populations analyzed in the present study and also in those Italian populations samples reported in the literature (**Tables 1** **and** **2**). Population samples were also grouped in main regions (North, Central, South, West, and East) in order to investigate the role of geography in the distribution of mtDNA variation.

**Table 1:** Diversity indices computed for different Italian regions based on HVS-I data (sequence segment 16090–16365).

*Population*	*Region*	*Pop ID*	*Reference*	*N*	*k*	*k/n*	*S*	*h*	Π	*M*
Liguri	NW	1	p.s.	50	40	0.8	53	0.962±0.021	0.01426±0.0145	4.875
Torino	NW	5	[84]	50	45	0.9	49	0.993±0.007	0.01483±0.0011	5.056
Ladin	NE	2, 13	p.s. [22], [62], [63], [66]	504	170	0.3	106	0.960±0.005	0.01251±0.0004	4.252
Pavia	NE	6	[84]	47	35	0.7	44	0.969±0.017	0.01316±0.0012	4.502
Udine	NE	3	p.s.	51	32	0.6	38	0.903±0.038	0.01231±0.0135	4.19858
Arezzo/Chiusi	CW	1	[9]	14	14	1	22	1.000±0.027	0.01488±0.0129	5.088
Casentino	CW	15	[8]	122	77	0.6	167	0.979±0.007	0.02409±0.0082	8.190
Collevecchio/Magliano Sabino	CW	3	[9]	12	11	0.9	14	0.985±0.040	0.01201±0.0015	4.106
Elba	CW	2	[9]	16	6	0.4	11	0.683±0.120	0.00853±0.0017	2.908
Firenze	CW	9	[84]	48	40	0.8	54	0.980±0.014	0.01332±0.0012	4.556
Jenne	CW	22	[85]	103	34	0.3	47	0.834±0.036	0.01006±0.0360	3.440
Latini	CW	5	p.s.	48	29	0.6	35	0.902±0.039	0.01003±0.0010	3.429
Latium	CW	20	[86]	52	37	0.7	48	0.959±0.019	0.01313±0.0014	4.492
Murlo	CW	16	[8]	86	60	0.7	68	0.976±0.010	0.01327±0.0009	4.524
Roma	CW	12	[84]	58	49	0.8	55	0.987±0.008	0.01433±0.0011	4.901
Terni	CW	11	[84]	29	20	0.7	33	0.941±0.034	0.01201±0.0014	4.108
Tuscany	CW	4	[9], [10], [87]	127	86	0.7	77	0.982±0.007	0.01305±0.0075	4.464
Vallepietra	CW	21	[85]	21	8	0.4	17	0.871±0.044	0.01281±0.0014	4.381
Volterra	CW	14	[8]	114	57	0.5	62	0.955±0.013	0.01193±0.0007	4.057
Abruzzo	CE	17	[86], [88]	61	53	0.8	62	0.990±0.007	0.01500±0.0010	5.131
Ancona	CE	10	[84]	73	55	0.7	59	0.963±0.017	0.01379±0.0010	4.717
Bologna	CE	7	[84], [89]	146	79	0.5	64	0.970±0.008	0.01250±0.0006	4.278
Center East	CE	23	[90]	83	62	0.7	60	0.974±0.012	0.01352±0.0009	4.625
Croatian Italians	CE	19	[86]	41	28	0.7	46	0.970±0.015	0.01524±0.0017	5.213
Modena	CE	8	[84]	44	33	0.7	43	0.958±0.023	0.01139±0.0012	3.895
Molise	CE	18	[86]	62	41	0.6	58	0.938±0.025	0.01260±0.0013	4.309
Piceni	CE	4	p.s.	53	43	0.8	56	0.985±0.009	0.01306±0.0011	4.414
Belvedere	SW	10	p.s.	50	41	0.8	44	0.980±0.013	0.01320±0.0010	4.532
Calabria	SW	27	[91], [92]	389	213	0.5	128	0.983±0.003	0.01521±0.0004	5.203
Campania	SW	30	[86]	48	41	0.8	59	0.980±0.014	0.01519±0.0014	5.166
Catania	SW	11	p.s.	40	35	0.9	45	0.990±0.010	0.01460±0.0012	4.979
Sicily	SW	28	[38], [93]–[96]	558	240	0.4	125	0.958±0.006	0.01289±0.0004	4.343
Trapani	SW	12	p.s.	40	30	0.7	36	0.977±0.013	0.01313±0.0013	4.465
Apulia	SE	26	[86]	26	24	0.9	43	0.991±0.015	0.01550±0.0022	5.304
Basilicata	SE	25	[91]	92	65	0.7	70	0.983±0.007	0.01290±0.0008	4.428
Grecani Salentini	SE	8	p.s.	47	37	0.8	44	0.989±0.007	0.01310±0.0011	4.480
Lucera	SE	6	p.s.	60	42	0.7	55	0.976±0.011	0.01345±0.0011	4.586
South Apulia	SE	9	p.s.	53	38	0.7	49	0.973±0.014	0.01579±0.0010	5.401
Sanniti	SE	7	p.s.	50	41	0.8	49	0.988±0.008	0.01420±0.0013	4.843
Sardinia	–	29	[38], [87], [97]	351	171	0.4	98	0.950±0.009	0.01183±0.0004	4.033
**Geographical region**										
North Italy	–	–		702	267	0.4	126	0.963±0.004	0.01282±0.0004	4.295
Central Italy	–	–		1413	500	0.4	216	0.958±0.004	0.01243±0.0002	4.113
South Italy	–	–		1453	569	0.4	183	0.973±0.002	0.01368±0.0002	4.541
West Italy (without Sicily)	–	–		1437	578	0.4	232	0.969±0.003	0.01315±0.0002	4.405
West Italy (with Sicily)	–	–		2075	709	0.3	236	0.963±0.003	0.01260±0.0002	4.133
East Italy	–	–		1493	520	0.3	165	0.964±0.003	0.01277±0.0002	4.200

NW = North-West; NE = North-East; CW = Center-West; CE = Center-East; SW = South-West; SE = South-East; *N* = sample size; k = number of different haplotypes; S = segregating sites; h = haplotype diversity; π = nucleotide diversity; M = average number of nucleotide differences.

**Table 2:** Diversity indices computed for different Italian regions based on Y-SNPs.

*Population*	*Region*	*Reference*	*N*	*k*	*k/n*	*Gene Diversity*
Liguria	NW	Present study	46	9	0.19	0.7662±0.0502
Ladin	NE	[14]	34	6	0.17	0.5348±0.0979
Udine	NE	Present study	47	10	0.21	0.7761±0.0441
Central Tuscany	CW	[14]	40	8	0.20	0.7397±0.0616
Elba Island	CW	[14]	94	7	0.07	0.6742±0.0445
Latini	CW	Present study	44	11	0.25	0.8256±0.0395
Latium	CW	[14]	43	9	0.20	0.8026±0.0388
Tuscany-Latium border	CW	[14]	76	7	0.09	0.7554±0.0350
Central Marche	CE	[14]	59	7	0.11	0.7294±0.0364
Marche	CE	[11]	162	13	0.08	0.8489±0.0152
Marche-Appennine	CE	[14]	25	7	0.28	0.8033±0.0514
Modena	CE	[98]	62	8	0.12	0.5320±0.0743
Piceni	CE	Present study	38	9	0.23	0.8208±0.0450
Rimini-Val Marecchia	CE	[99]	163	12	0.35	0.6990±0.0308
Belvedere	SW	Present study	27	9	0.33	0.8547±0.0477
East Campania	SW	[14]	46	7	0.15	0.6870±0.0618
Sicily	SW	Present study	57	12	0.21	0.8327±0.0311
West Campania	SW	[14]	80	10	0.12	0.8446±0.0224
West Calabria	SW	[14]	57	7	0.12	0.7525±0.0307
Sanniti	SE	Present study	30	10	0.33	0.8644±0.0409
Grecani Salentini	SE	Present study	47	7	0.14	0.8122±0.0242
Lucera	SE	[31]	60	9	0.15	0.8365±0.0236
South Apulia	SE	[14]	49	9	0.18	0.8529±0.0237
Sardinia		[100]	336	14	0.04	0.8098±0.0136
**Geographical region**						
North Italy	–	–	127	14	0.11	0.8400±0.0189
Central Italy	–	–	806	21	0.03	0.8870±0.0053
South Italy	–	–	453	20	0.04	0.8909±0.0060
West Italy (without Sicily)	–	–	553	17	0.03	0.8567±0.0094
West Italy (with Sicily)	–	–	610	20	0.03	0.8705±0.0078
East Italy	–	–	776	22	0.02	0.9034±0.0037

Codes are as in Table 1.

Mitochondrial DNA haplotypes for the samples analyzed in the present study are reported in **Table S3**. **Table 1** shows the molecular diversity values based on mtDNA data for 41 Italian population samples. The values indicate that the Isle of Elba is, by far, the Italian population sample that shows the lowest diversity for all the indices computed, probably as a consequence of its relative isolation from the country. It has been reported that this was a well-known enclave of Etruscan influence, and some mtDNA particularities have been described before [8], [9]. Alternatively, low molecular diversity could be due to low sample sizes, although this fact is mirrored in the standard deviation of the different estimates. Excluding the Isle of Elba, haplotype diversity in Italy ranges from 0.834 to 1, nucleotide diversity from 0.01003 to 0.02409, and the average value of nucleotide differences from 3.4 to 8.19 (a value that is correlated with the nucleotide diversity). In general, Italy shows some level of heterogeneity when examined for diversity values.

When grouping populations by main geographical regions, it can be observed that Central Italy has slightly lower values than North and South Italy for all the indices computed (**Table 1**). The higher diversity values were found in South Italy. Diversity values are however very similar when examining populations located in West Italy *versus* those in the East. The inclusion of Sicily (as part of South Italy) in the computation does not substantially change these estimates (**Table 1**).

Y-SNP data were obtained for all the samples analyzed in the present study (**Table S4**). **Table 2** shows the diversity indices for the Y-SNPs in different Italian populations. The Y-STR diversity values for the samples analyzed in the present study and other Italian and European samples have already been reported in Brisighelli et al. [33]. As expected, diversity values of Y-SNP haplogroup patterns are lower than those obtained for the mtDNA haplotypes given that the indices are based on haplogroup and not on Y-STR haplotypes. In fact, values based on Y-STR profiles (minimum or extended Yfiler profiles) [33] are higher than those observed for the HVS-I profiles. Ladins are among the populations with the lowest Y-SNP diversity values, while the Grecani Salentini show diversity values that are comparable to other Italian samples. Modena shows remarkable low haplotype diversity values.

### Phylogeography

The mtDNA haplogroup make-up of Italy as observed in our samples fits well with expectations in a typical European population. Thus, most of the Italian mtDNAs (∼89%) could be attributed to European haplogroups H (∼40%), I (∼3%), J (∼9%), T (∼11%), U (∼20%; U minus U6), V (∼3%), X (∼2%) and W (∼1%); **Figure 1**. There are however important differences in haplogroup frequencies when examining them by main geographical regions. Thus, for instance, haplogroup H is 59% in the North, 46% in the Center, and decays to ∼33% in the South; moreover, these regional differences are statistically significant: North *vs* South (Pearson's chi-square, unadjusted-*P* value<0.00003), and Center *vs* South (Pearson's chi-square, unadjusted-*P* value<0.03724).

Mitochondrial DNA haplotypes of African origin are mainly represented by haplogroups M1 (0.3%), U6 (0.8%) and L (1.2%); from here onwards, L will be used to refer to all mtDNA lineages, excluding the non-African branches N and M [60], [61].

**Figure 3:**
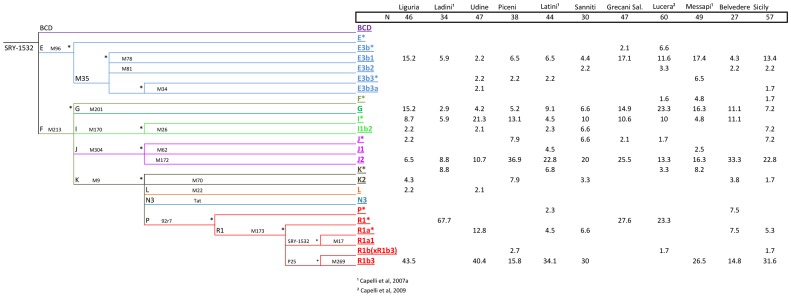
Phylogeny of Y-chromosome SNPs and haplogroup frequencies in different Italian populations.

A total of 282 Y-chromosomes were analyzed for a set of Y-SNPs and were classified into 22 different haplogroups (**Figure 3**). Two haplogroups were not found, even though markers defining these clades were tested: N3 and R1a1. Five haplogroups represented 76.71% of the total chromosomes: R1b3, J2, I(xI1b2), E3b1 and G. The frequencies averaged across populations were 26%, 21.2%, 10.2%, 9.9% and 9.2%, respectively. The remaining haplogroups sum to 23.2% in the total sample, and never above 4% in single population samples.

R1b3 frequency was found to be higher in the northern part of the country, while the Y-chromosome haplogroups G and E3b1, J2 and I(xI1b2)frequencies were higher in the south and in the central part of the country, respectively (**Figure 1**).

Regional differences are substantially higher in the Y-chromosome than in the mtDNA. Thus, for instance, haplogroup R in the Y-chromosome was 54% in the North, 18% in the Center, and 31% in the South. Frequency differences were statistically significant between North *vs* Center (Pearson's chi-square, unadjusted-*P* value = 0.0014), and North *vs* South (Pearson's chi-square, unadjusted-*P* value<0.00004). Haplogroup J2 also revealed important regional differences; it added to 9% in the North, 37% in the Center, and 22% in the South, with statistically significant differences between the North *vs* Center (Pearson's chi-square, unadjusted-*P* value<0.00002), North *vs* South (Pearson's chi-square, unadjusted-*P* value<0.00148), and in the limit of significance Center *vs* South (Pearson's chi-square, unadjusted-*P* value<0.049).

### Autosomal ancestry in Italy

A panel of 52 AIMs was genotyped in 435 Italian individuals in order to estimate the proportion of ancestry from a three-way differentiation: sub-Saharan Africa, Europe and Asia. Structure analyses allowed us to infer membership proportions in population samples, and these proportions can be graphically displayed, as in **Figure 2**. This analysis indicated that Italians have a basal proportion of sub-Saharan ancestry that is higher (9.2%, on average) than other central or northern European populations (1.5%, on average). The amount of African ancestry in Italians is however more comparable to (but slightly higher than) the average in other Mediterranean countries (7.1%). **Figure 2** shows in a triangle plot the relationships of Italians compared to other European, African and Asian populations.

PCA observations confirmed the results from Structure analysis, clustering Italian profiles tightly with other European ones. Thus, PCA indicated that North, Central and South Italy do not show differences between them, nor from other European populations (**Figure 2**). PCA also indicated clear-cut differences between Italians, Africans and Asians (**Figure 2**).

### AMOVA

AMOVA analyses were carried out following different grouping schemes. The samples were pooled into a single population, but also by considering main Italian regions. Analyses were carried out over haplogroups and haplotypes of the Y-chromosome and the mtDNA (**Table 3**).

**Table 3:** AMOVA analysis of main Italian regions (Permutations: 20000; *P*-value<0.0000) for the mtDNA control region data and the Y-chromosome STRs and SNPs.

	All populations (%)	North vs Center vs South (%)	West vs East (%)
**HAPLOTYPES**			
**mtDNA (48 populations)**			
Among pops	0.79	0	0
Within pops	99.21	99.25	99.21
Among pops within groups	–	0.75	0.79
**Y-chromosome (15 populations)**			
Among pops	2.34	1.18	0
Within pops	97.66	97.32	97.85
Among pops within groups	–	1.50	2.15
**HAPLOGROUPS**			
**mtDNA (19 populations)**			
Among pops	1.17	0.36	0
Within pops	98.83	98.72	98.83
Among pops within groups	–	0.92	1.17
**Y-chromosome (24 populations)**			
Among pops	13.92	0.07	0.83
Within pops	86.08	86.06	85.74
Among pops within groups	–	13.87	13.44

Sardinians were not included in the analysis. References for population samples are given in Table S2.

AMOVA indicated that, among populations, variance was more strongly stratified for the Y-chromosome than for the mtDNA; the difference was much more marked for the analysis based on haplogroups (14.39% *vs* 1.17%) than for the analysis based on haplotypes (2.34% *vs* 0.79%). Among population variance was very low when analyzing main geographical regions; however, it was the latitude (North *vs* Center *vs* South) that appeared to account for higher values of among-population variance rather than longitude (West *vs* East), with the exception of the Y-chromosome haplogroups (although the values are below 1%); **Table 3**. Again, the Y-chromosome showed slightly higher values of among-population variance than did the mtDNA. For the Y-chromosome, a significant proportion of the within-population variance moved to among-population within-groups variance, probably due to the fact that all population samples had a very high proportion of singleton Yfiler haplotypes, elevating the maximum values of haplogroup diversity for all of them [33].

### Linguistic isolates: Ladin and Grecani Salentini

Two linguistic isolates are represented in the samples analyzed in the present study: the Ladin and the Grecani Salentini.

Other population samples of the Ladin have already been analyzed in the literature [22], [62], [63]. We here sampled 41 new individuals from the locality of Val Badia. As reported in **Table 4** for the mtDNA, Val Badia Ladins showed relatively high nucleotide diversity patterns compared to other Ladin populations, but intermediate haplotype diversity values. Compared to other Italian populations, diversity in Ladin populations is generally lower (**Table 1**). For Y-chromosome haplogroups, the differences between Ladin and the rest of Italy were more evident, with the Ladin showing much lower values than average Italians.

**Table 4:** Mitochondrial DNA molecular diversity values of different Ladin populations.

Ladin populations	References	*N*	*k*	*k/n*	*S*	*h*	Π	*M*
Val Badia	p.s, [22]	97	55	0.6	60	0.958±0.012	0.01206±0.0008	4.101
Val Gardena	[22], [66]	56	27	0.5	42	0.906±0.027	0.01216±0.0011	4.158
Colle S. Lucia	[62], [66]	30	17	0.8	33	0.947±0.022	0.01885±0.0010	6.448
Val di Fassa	67	47	25	0.5	34	0.932±0.026	0.01242±0.0012	4.135

**Figure 4:**
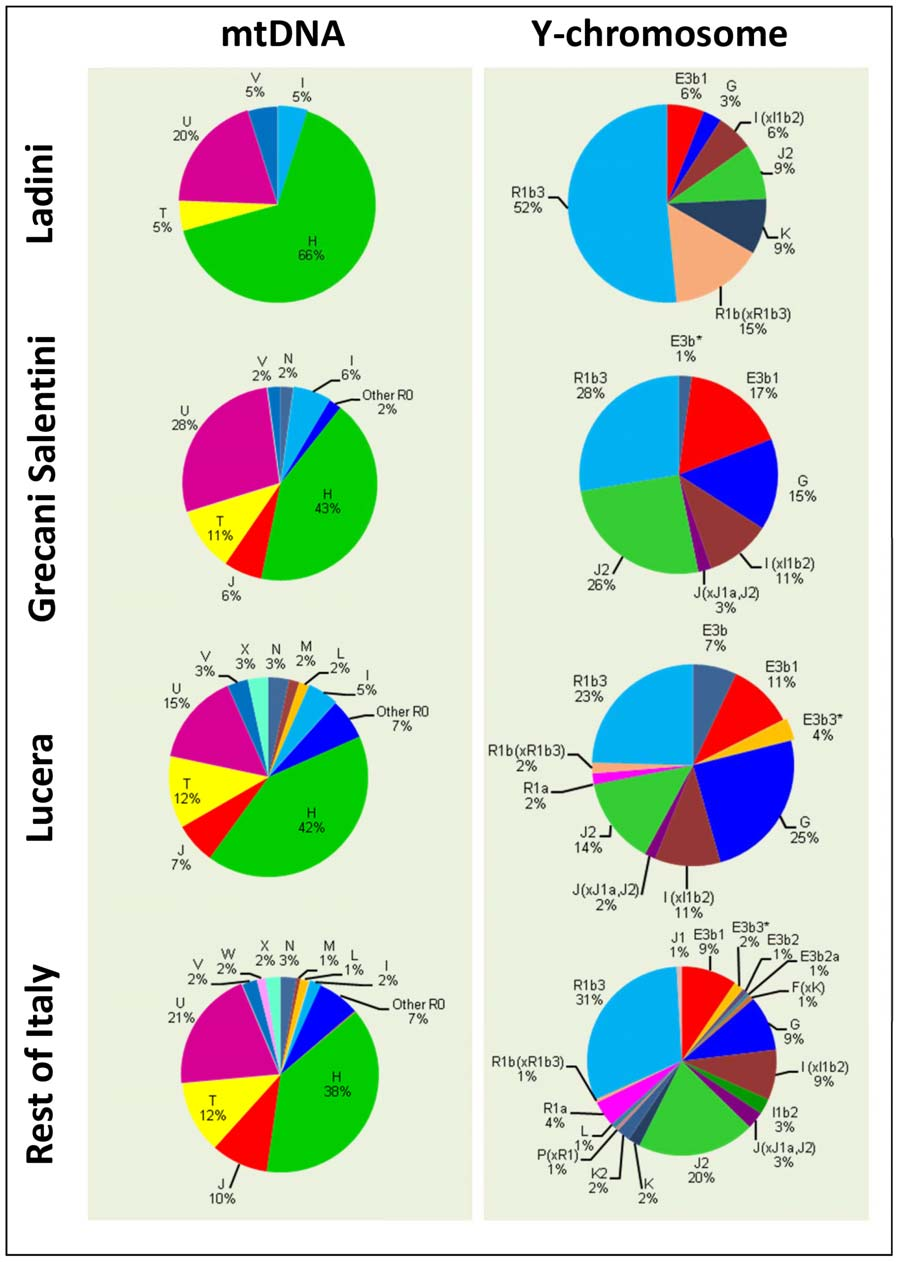
Haplogroup frequencies of Ladins, Grecani Salentini and Lucera compared to the rest of the Italian populations analyzed in the present study.

The differences between Ladin and other populations were more evident when examining haplogroup frequency patterns (**Figure 4**). The frequency of haplogroup H (58%) was above the frequency of H in North Italy (55%), and was extremely high (58%) compared to the average for Italy (38%) (Pearson's Chi-square test, *P*-value = 0.0005). While haplogroup U was found to have approximately the same frequency as other Italian populations, haplogroup T was 5% compared to 12% in Italy generally (7% in the North). Other differences were apparent, but sample sizes were relatively low to yield significant statistical differences.

Differences are more important when examining Y-chromosome haplogroup frequencies. R1b3 reached 52% in Ladin populations but only 31% in the general population, and also in the North (Pearson's Chi-square test, *P*-value = 0.0087); **Figure 4**. More remarkable are the differences when considering the remaining R1b lineages, that is, R1b(xR1b3), which account for 15% of the lineages in Ladins, but only for 1% in the general population (Pearson's Chi-square test, *P*-value = 0.0001). Other haplogroups showed substantial haplogroup differences (e.g. J2) but the sample size was again too small.

Due to the availability of data for mtDNA in several Ladin communities, we were able to carry out an AMOVA analysis in order to investigate the level of population stratification in these communities. The data indicated that among-population variance is 1.09%, a value that is therefore higher than the average for the Italian Peninsula (0.79%; **Table 4**).

Some interesting features were also found for Ladin populations when examined at the haplotype level. For instance, the HVS-I profile G16129A C16192T A16270G T16304C was found in four Ladins from Val Badía; this profile belongs to haplogroup U5b3f [20]. In a large in-house database of worldwide profiles (>130,000 HVS-I segments), this sequence was only found sporadically in other Italian regions and in Spain (Catalonia, Galicia, and Ibiza in the Balearic Islands). U5b3f is a minor clade of U5b3, the only haplogroup reported to date that has been found to represent the glacial refuge zone in Northern Italy and a source population for human re-colonization of the continent at the beginning of the Holocene. The study of Pala et al. [20] indicates that this lineage mainly expanded along the Mediterranean coast towards the Iberian Peninsula; one sub-clade also reached Sardinia 7000–9000 years ago. The branch observed in the Ladins is younger and could also have participated in the Mediterranean spread of U5b3f towards Iberia, given its presence in modern-day Spain. The data suggest that the U5b3f members observed in the Ladins probably predate the Ladin ethnogenesis and, given that this population has somehow become isolated from other neighboring populations, could reach a substantial frequency in some other Ladin communities, as is the case for the Val Badia. Another example is the U3 profile A16233G C16256T T16311C A16343G, which was only found in five Ladins from Alto Adige (Val Badia and Val Gardena), while T16352C C16354T was found in six individuals from Val Badia in South Tyrol.

Diversity values in the Grecani Salentini samples were similar to those observed in other Italian regions. Moreover, they also show haplogroup frequency patters in the Y-chromosome and the mtDNA that matches well with other Italian samples. The haplogroups are typically European (**Figure 4**); given the southern location of the Grecani Salentini in the Italian Peninsula, it is noticeable that there is no evidence of North African lineages. Note however, that at higher level of phylogenetic resolution, there are signals on the Y-chromosome of North African enrichment in South Italy [31].

### The North African historical legacy in South Italy

We sampled 60 individuals from Lucera. This population sample showed diversity values that fell within the average of a typical Italian population, regarding the mtDNA (**Table 1**) and the Y-chromosome (**Table 2**). Additionally, at the level of haplogroup frequencies, Lucera matched well with other Italian populations (**Figure 4**).

There are two mtDNA haplogroups, namely U6 and M1 that can be considered to be of North African origin and could therefore be used to signal the documented historical input of this African region into Lucera. In our full set of samples, we observed five U6 haplotypes belonging to sub-haplogroups U6a, U6a2, and U6a4. Only one of these haplotypes was observed in Lucera. However, the other three U6 haplotypes were observed in the vicinity of the population of South Apulia, and another at the tip of the Peninsula (Calabria). Regarding M1 haplotypes, we observed only two carriers in our samples sharing the same HVS-I haplotype; both were found in Trapani (West Sicily).

Therefore, while South Italy shows evidence of having female introgression from North Africa, this African influence seems not to be particularly centered in the Lucera. In the Y-chromosome, we did not observe any signal of North African introgression; at least, no more than for other regions of Italy (perhaps with the exception of Sicily [31]). This again contrasts with the results of previous studies based on the Y-chromosome (but at higher or different level of phylogenetic resolution involving the genotyping of African minor sub-lineages) where signals of North African influence were observed at this latitude of the Peninsula [31].

## Discussion

A meta-analysis of Y-chromosome and mtDNA sequence data was undertaken in order to investigate patterns of genetic variation throughout Italy. Molecular indices indicated that most of the Italian samples show diversity values that are comparable to other European populations. However, some differences were shown to exist, especially in isolated Ladin populations. Regional differences were much more evident when examining haplogroup frequencies in both uniparental markers. The differences were again more remarkable for the two linguistic isolates, the Ladins and Grecani Salentini. AMOVA also indicated the existence of significant population stratification along the length of the country, which appeared more remarkable for the Y-chromosome and for haplogroups than for haplotypes. These figures have however to be considered with caution given the different mutability of the markers being analyzed [64]; see also a discussion in [65].

Over the last few years, the interest in genetically isolated populations has increased, especially in biomedical studies, where there exists a growing interest in revealing genetic variants associated to disease. Genetic isolates generally originate as a result of group “foundation” by a small number of individuals presenting initially low variability. We have here analyzed a new sample of the Ladins, a well-known linguistic and genetic isolate from the Italian Alps. Some investigations were focused on the Ladin Romance speaking populations, distributed between Trentino, the Veneto regions and South Tyrol area [22], [62], [63], [66]. As also observed in the present study, Ladin communities show marked genetic differentiation with neighboring (non-Ladin) populations. Differences were also observed between the different Ladin groups; for instance, AMOVA analysis also indicated that the different Ladin communities show a level of population stratification that is higher than the average in the rest of Italy. These results are also consistent with the recent study by Coia et al. [67], derived from micro-geographical analysis of nine sample populations from Trentino (Eastern Italian Alps). Genetic differences between Ladin samples are most likely to be due to the limited historical gene flow existing between these communities [22]. In this regard, it is also noticeable that, while the South Tyrol populations show clear signatures of isolation, the Veneto groups presented a high degree of genetic variability [68].

The Grecani Salentini also showed signatures of genetic isolation when compared to other Italian populations, but the differences are not as marked as observed for the Ladins. The differences with respect to neighboring Italian populations were not evident when observing individual haplotypes (as occurs with the Ladins), but were clearer when considering haplogroup frequencies (**Figure 4**). Larger sample sizes are needed in order to gather more signatures about the demographic past of this population. Thus, the Ladins show a more distinctive pattern than the Grecani Salentini, which is to be expected given that not only is the Ladin population a linguistic isolate, but also that these communities are confined to isolated geographical areas of the Alps.

Apart from the regional and local genetic differences observed in Italy, it is also worth examining global genetic patterns along the length of continental Italy.

Geographical clines of Y-chromosome haplogroups in Europe have been previously reported in the literature [13]; these patterns have found support in archaeological and linguistic evidence. In the Italian peninsula, the Y-chromosome variation also shows a clinal pattern along the North–South axis; the Mesolithic haplogroup R1*(xR1a1) shows higher frequency in the North while the Neolithic haplogroup J2-M172 is superposed to this Mesolithic strata with frequency patterns running in the opposite direction [14], [69]. The results of the present study agreed with these earlier findings. Thus, for instance, R1b3 reached 31% in the North, 16% in the Center, and 14% in the South. Frequency of Y-chromosome haplogroup J2 was found to be 9% in the North, 37% in the Center, and 22% in the South (average in Italy: 14.5%). Haplogroup J2 is widely believed to be associated with the spread of agriculture from Mesopotamia. The main spread of J2 into the Mediterranean area is thought to have coincided with the expansion of agricultural populations during the Neolithic period. As reported by Di Giacomo et al. [12], haplogroup J “…*constitutes not only the signature of a single wave-of-advance from the Levant but, to a greater extent, also of the expansion of the Greek world, with an accompanying novel quota of genetic variation produced during its demographic growth…*”; also that “*…in the central and west Mediterranean, the entry of J chromosomes may have occurred mainly by sea, i.e., in the south–east of both Spain and Italy…*”. J2-M12 is almost totally represented by its sublineage J2-M102, which shows frequency peaks in both the southern Balkans and north-central Italy (14%; [13]). J2-M67 is most frequent in the Caucasus, and J2-M92 indicates affinity between Anatolia and southern Italy (21.6%; [13]). For the J1-M170 clade, the peaks of J1-M267 are in the Levant and in northern Africa, and it is closely associated to the diffusion of the Arab people, dropping abruptly outside of this area (including Anatolia and the Iberian peninsula), even if it shows an appreciable percentage in Sicily [70]. In a recent study, Pala et al. [71] confirmed that mtDNA haplogroups J and T and their major sub-clades (J1 and J2, T1 and T2) most likely arose in the Near East at the time of the first settlement by modern humans and the LGM. These haplogroups started to spread from the Near East into Europe immediately after the peak of the last glaciation, about 19 kya ago, with a major expansions in Europe in the Late Glacial period, about 16–12 kya ago, thus indicating that many of the Neolithic expansions from southern Europe into Central Europe and the Mediterranean might have been indigenous dispersal of these lineages.

Latitudinal clinal frequency patterns are also observed for the mtDNA haplogroups mirroring those of the Y-chromosome. As reported by Richards et al. [38], haplogroups H, K, T*, T2, W, and X are the major contributors to the Late Upper Paleolithic, and the central-Mediterranean region has the greatest Middle Upper Paleolithic component outside the Caucasus. In agreement with the Y-chromosome, we observed that all these Paleolithic haplogroups together add to approximately 70.3% in the North, 60.8% in the Center, and 54% in the South of Italy. The opposite pattern was observed for the main mtDNA Neolithic component, represented by haplogroups J and T1, which accounted for 5.8% in the North, 10.3% in the Center, and 14.1% in the South (Italian average: 10.5%).

As early as 1934, [72], Vere Gordon Childe suggested that the indigenous communities of hunters and gatherers of the Mesolithic European cultures were replaced by communities of farmers migrating to the North from the Middle East, a process that lasted for several generations. The first stream of emigration followed the route along the continental Balkan Peninsula and the Danube, while another, slightly later, emigration spread along the coasts of the Mediterranean Sea from East to West. The latter path would fit well with the distribution of other Neolithic cultural features, such as the so-called Cardium Pottery (or Cardial Ware) [73], the ceramic decorative style that better defines the Neolithic culture. This culture entered from Greece towards the South-Center of Italy through the Adriatic Sea, carried by the same farmers that introduced, for instance, Y-chromosome haplogroup J2 at about the same frequency in Central and South Italy, but with lower introgression into the North; from here followed further Mediterranean expansions towards Iberia.

The sub-clade E3b1 (probably originating in eastern Africa) has a wide distribution in sub-Saharan Africa, Middle East and Europe. This haplogroup reaches a frequency of 8% in the North and Center and slightly higher in the South of Italy, 11% (**Figure 1**). It has also been argued that the European distribution of E3b1 is compatible with the Neolithic demic diffusion of agriculture [15]; thus, two sub-clades, E3b1a- M78 and E3b1c-M123 present a higher occurrence in Anatolia, the Balkans and the Italian peninsula. Another sub-clade, E3b1b-M81 is associated with the Berber populations and is commonly found in regions that have had historical gene flow with Northern Africa, such as the Iberian peninsula [74], [75]–[76]–[78], including the Canary Islands [75], and Sicily [70], [79]; the absence of microsatellite variation suggests a very recent arrival from North Africa [80]. If we assume that all E3b1 represents the only Y-chromosome continental African contribution to Italy and L and U6 lineages the continental African mtDNA component, the African component in Italy is higher for the Y-chromosome (8–11%) than for mtDNA (1–2%). The origin of sub-Saharan African mtDNAs in Europe (including Italian samples) has been recently investigated by Cerezo et al. [81]; the results indicate that a significant proportion of these lineages could have arrived in Italy more than 10,000 years ago; therefore, their presence in Europe does not necessarily date to the time of the Roman Empire, the Atlantic slave trade or to modern migration.

In addition, the Northern African influence in the Italian Peninsula is evidenced by the presence of Northern African Y chromosome haplogroups (E1-M78) in three geographically close samples across the southern Apennine mountains: East Campania, Northwest Apulia and Lucera [31]. The Lucera sample analyzed in the present study did not however show a higher impact from North Africa than for other areas from southern Italy [31].

Finally, in agreement with uniparental markers, analysis of AIMs as carried out in the present study indicated that Italy shows a very minor sub-Saharan African component that is, however, slightly higher than non-Mediterranean Europe. This agrees with the recent findings of Cerezo et al. [82] based on the analysis of entire mtDNA genomes pointing to the arrival in ancient and historical times of sub-Saharan African people to the Mediterranean Europe, followed by admixture.

The present study represents the largest meta-analysis carried out to date for the Italian peninsula. We observed that the Y-chromosome and the mtDNA retain the imprint of the major ancestral events occurring in Italy; however, the Y-chromosome shows more marker regional differences than does the mtDNA. It is difficult to infer what proportion of these differences can be attributed not only exclusively to gender demographic differences, but also to the fact that both markers were analyzed to different levels of molecular resolution. Italy shows clines of variation attributable to the demographic movements of the first Paleolithic settlements, posteriorly modeled by the Mesolithic and, to a lesser extent, Neolithic farmers. Regional differences arose with time, which are more notable in linguistic isolates, such as the Ladin populations, and to a minor extent, the Grecani Salentini. Lot of effort has been dedicated during the last two decades to the study of Italian populations. Further studies are needed in order to dig into some of the many demographic movements occurring in the Italian peninsula along history. Entire genome sequencing of particular lineages (in the line of e.g. [20]) and nuclear DNA genomic studies are needed in order to explore hypothesis beyond what has been done to date in Italy.

## Supporting Information

Table S1 **mtSNPs and primers used to characterize J/T and U and some of their sub-clades.**
(XLS)

Table S2 **References to the population samples used in the present study for population comparison analysis.**
(XLS)

Table S3 **Mitochondrial DNA control region haplotypes obtained from the samples analyzed in the present study.**
(XLS)

Table S4 **Y-SNP and Y-STR profiles of the Italian samples analyzed in the present study.** Note that the Y-STR data has already been reported in Brisighelli et al. [65].
(XLS)
