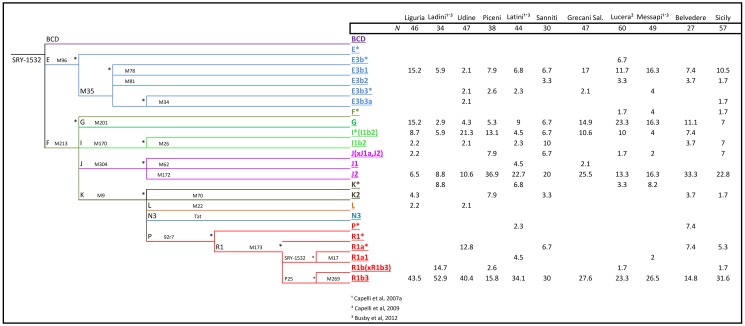# Correction: Uniparental Markers of Contemporary Italian Population Reveals Details on Its Pre-Roman Heritage

**DOI:** 10.1371/annotation/ea14adcb-033d-492d-8f8b-e047aa080cd4

**Published:** 2014-01-17

**Authors:** Francesca Brisighelli, Vanesa Álvarez-Iglesias, Manuel Fondevila, Alejandro Blanco-Verea, Ángel Carracedo, Vincenzo L. Pascali, Cristian Capelli, Antonio Salas

Several errors occurred in the data within the third and fifth paragraphs of the section "Phylogeography", and the third and fourth paragraphs within "Linguistic isolates: Ladin and Grecani Salentini" under the Results section. Please see the corrected paragraphs, with updated haplogroup percentages below:

Phylogeography:

A total of 282 Y-chromosomes were analyzed for a set of Y-SNPs and were classified into 22 different haplogroups (Figure 3). Two haplogroups were not found, even though markers defining these clades were tested: N3 and R1a1. Five haplogroups represented 76.71% of the total chromosomes: R1b3, J2, I(xI1b2), E3b1 and G. The frequencies averaged across populations were 31%, 19%, 8.3%, 10.2% and 11.2%, respectively. The remaining haplogroups sum to 18.6% in the total sample, and never above 2.7% in single population samples.

Regional differences are substantially higher in the Y-chromosome than in the mtDNA. Thus, for instance, haplogroup R in the Y-chromosome was 53% in the North, 29.3% in the Center, and 30% in the South. Frequency differences were statistically significant between North vs Center (Pearson’s chi-square, unadjusted-P value = 0.0014), and North vs South (Pearson’s chi-square, unadjusted-P value < 0.00004). Haplogroup J2 also revealed important regional differences; it added to 9% in the North 29.3% in the Center, and 20.7% in the South, with statistically significant differences between the North vs Center (Pearson's chi-square, unadjusted-P value<0.00002), North vs South (Pearson's chi-square, unadjusted-P value<0.00148), and in the limit of significance Center vs South (Pearson's chi-square, unadjusted-P value<0.049).

Linguistic isolates: Ladin and Grecani Salentini:

The differences between Ladin and other populations were more evident when examining haplogroup frequency patterns (Figure 4). The frequency of haplogroup H (66%) was above the frequency of H in North Italy (55%), and was extremely high (66%) compared to the average for Italy (38%) (Pearson’s Chi-square test, P-value = 0.0005). While haplogroup U was found to have approximately the same frequency as other Italian populations, haplogroup T was 5% compared to 12% in Italy generally (7% in the North). Other differences were apparent, but sample sizes were relatively low to yield significant statistical differences.

Differences are more important when examining Y-chromosome haplogroup frequencies. R1b3 reached 53% in Ladin populations but only 31% in the general population, and also in the North (Pearson's Chi-square test, P-value = 0.0087); Figure 4. More remarkable are the differences when considering the remaining R1b lineages, that is, R1b(xR1b3), which account for 15% of the lineages in Ladins, but only for 2% in the general population (Pearson's Chi-square test, P-value = 0.0001). Other haplogroups showed substantial haplogroup differences (e.g. J2) but the sample size was again too small.

Figures 1 and 3 have been updated accordingly. Please view the corrected images below:

Figure 1: 

**Figure:**
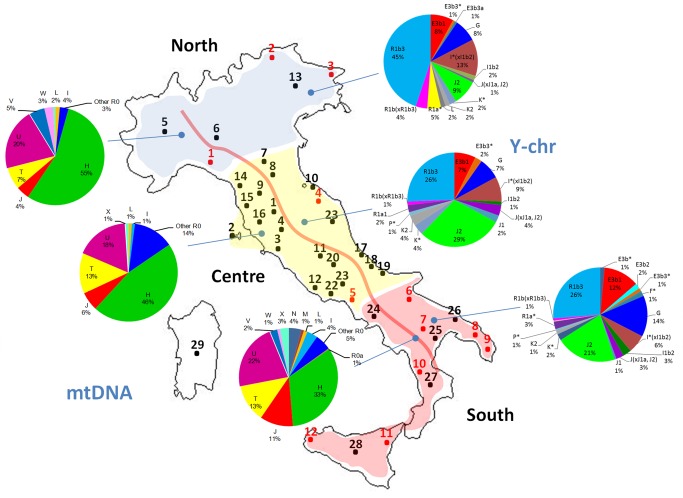


Figure 3: 

**Figure:**